# Group-level test–retest reliability assessment using systemic physiology augmented functional near-infrared spectroscopy during a passive-listening task

**DOI:** 10.1117/1.NPh.13.1.015005

**Published:** 2026-01-20

**Authors:** Abigail A. Mollison, Eric R. Rodriguez, Abigail Metzger, Maureen J. Shader

**Affiliations:** Purdue University, Department of Speech, Language, and Hearing Sciences, West Lafayette, Indiana, United States

**Keywords:** functional near-infrared spectroscopy, systemic-physiology augmented functional near-infrared spectroscopy, test–retest reliability, systemic-physiology, hemodynamic signal denoising, optode digitization

## Abstract

**Significance:**

Functional near-infrared spectroscopy (fNIRS) is a valuable neuroimaging technique for auditory-research tasks. However, fNIRS data are influenced by systemic-physiological processes, resulting in noisier signal quality that may compromise test–retest reliability. fNIRS reliability may also be influenced by changes in cap placement across sessions.

**Aim:**

We investigated the effectiveness of systemic-physiology denoising methods on the test–retest reliability of group-level fNIRS data during a passive-listening task.

**Approach:**

Fifteen participants completed two identical sessions of a passive-listening task; source-detector optode locations were digitized for each session. Different denoising methods were compared to investigate the effect of systemic-physiology correction on the consistency of across-session hemodynamic response amplitudes. Test–retest reliability for each method was assessed using the intraclass correlation coefficient (ICC). Changes in optode and channel locations across sessions were calculated to track shifts in cap placement.

**Results:**

Results revealed significant speech-evoked activity in bilateral auditory cortices following stimuli presentation. Group-level test–retest reliability metrics demonstrated good reliability across two identical test sessions. Accounting for the influence of systemic physiology resulted in improved reliability. Slight variations in optode placement did not significantly affect signal repeatability.

**Conclusion:**

This study supports the use of fNIRS for group-level task-evoked studies, demonstrating that systemic-physiology denoising methods can modestly improve test–retest reliability.

## 1 Introduction

Functional near-infrared spectroscopy (fNIRS) is a non-invasive neuroimaging modality that measures functional cortical activity in real-time under a variety of testing paradigms.^1^ fNIRS exploits the neurovascular coupling between cerebral blood flow and cortical neuronal activity to assess response to stimuli.^2^ Increased brain activity demands a greater oxygen supply, effectively adjusting the regulation of cerebral blood flow relative to activated brain regions.^2^ fNIRS operates by monitoring changes in the absorption of near-infrared light, which is used as a proxy to estimate the relative change in the amount of oxygenated and deoxygenated hemoglobin in the cortical blood.^3^ fNIRS systems are considerably cheaper, portable, and more accessible compared to functional magnetic resonance imaging (fMRI) systems, making fNIRS a viable alternative for functional neuroimaging measurements. In the interest of auditory research, fNIRS systems are advantageous as they are virtually silent, do not create any electromagnetic artifact, and are safe to use with hearing devices.^4^^,^^5^ Additionally, fNIRS provides relatively high resistance to speech-motion artifacts, which is of particular importance in auditory research, as participants are able to actively engage with and respond to stimuli.^6^ Participants are also able to sit upright while listening, allowing for more naturalistic testing conditions.

While both fMRI and fNIRS provide many advantages for *in vivo* research, additional factors can influence the signal captured during a task.^7^^–^^10^ These factors are present because live-monitoring of task-related brain activity captures systemic physiology alongside neural activity. Extracerebral blood flow and blood pressure in cerebral blood vessels are influenced by gross motor movement and by task type, which may elicit false positives in the estimated hemodynamic response.^2^^,^^11^ Additionally, changes in cortical activity directly correlate with and are influenced by changes in systemic physiology. However, neural activity can also influence changes in the circulatory system through task-linked activity, demonstrating the reciprocal nature of neurovascular coupling.^2^^,^^7^^,^^8^^,^^11^ Functional neuroimaging can also be influenced by systemic-physiology signals that are unrelated to blood flow or blood pressure, such as respiration, body temperature, and skin conductance, which can also fluctuate based on task-linked cortical activity.^7^^–^^10^ As such, fNIRS data are susceptible to between-subject and within-subject differences in systemic-physiological signals across multiple recordings.^12^ Even when completing an identical task in a subsequent fNIRS recording, the presence of systemic noise originating from the body may result in inconsistent data across testing sessions in the same participant or group of participants.^13^^–^^15^

Reduced within-subject test–retest reliability has limited the use of fNIRS in longitudinal studies, intervention studies, and other clinical applications. The presence of systemic-physiological and/or extracerebral noise likely contributes to this limitation in reliability.^16^ However, fNIRS can be made more reliable with physiology correction.^13^^,^^17^^,^^18^ Influence from physiological noise is primarily due to the nature of the delivery of near-infrared light signals.^7^^,^^11^ Infrared light photons are emitted through light sources placed on various locations on the scalp. This requires the photons to travel through extracerebral tissue before entering the brain, allowing for influence from extracerebral tissues (e.g., the scalp) on the signal being collected. One strategy for accounting for this extracerebral physiological influence is the use of short-separation channels/detectors, which improves signal quality and reliability in single-subject fNIRS recordings.^18^ The use of short channels is now considered a standard and necessary practice in fNIRS research.^1^ Short-channel detectors are positioned close to source optodes (∼8  mm separation in adults) to capture changes in the infrared light signal as it passes through only extracerebral tissue.^18^ In this way, short channels can be used to identify and remove systemic, non-neural hemodynamic changes from the extracerebral layers that could confound neural activity captured by the long channels (∼30  mm separation). While short-separation channels are considered essential to conducting fNIRS research, they are highly location-dependent and do not take individual differences in scalp thickness into account. Extracerebral hemodynamics are variable across the entire scalp and do not provide a comprehensive assessment of global systemic physiological signals that may be present in fNIRS data.^7^^,^^12^

A newer, more global method of noise reduction called systemic physiology augmented functional near-infrared spectroscopy (SPA-fNIRS) aims to address additional sources of noise through the removal of signals originating from systemic-physiological markers.^7^ SPA-fNIRS uses sensors attached to different points of the body to record various physiological signals (e.g., respiration rate, blood pressure, and skin conductance) in tandem with functional brain activity. SPA-fNIRS uses these multiple physiological signals to account for potential sources of noise. When used alongside short channels and within a test–retest paradigm, it is logical to assume that SPA-fNIRS may better account for confounding systemic physiological factors, and thus, result in more reliable data across multiple recording sessions.

Even after accounting for systemic and extracerebral noise, additional sources of variability may also be present in fNIRS data across sessions. Another important factor is the consistency in source-detector optode location (i.e., cap placement). As participants may be completing multiple studies across several days, weeks, or even years, the fNIRS cap ideally should be placed at the same precise position across sessions. Traditional fNIRS systems use multiple infrared light sources and detectors, typically arranged in a predetermined montage with specific locations coordinated with neural regions of interest. If the cap is placed inconsistently, it cannot be concluded that the activity recorded originates from the same region of interest for each subject in each session.^12^^,^^19^ Multiple efforts have been made to improve the consistency of optode placement, including guided placement using neuronavigational systems. Compared to standard approaches for cap placement (e.g., measured head circumference, ear-to-ear distance, and inion-to-nasion distance), formal guided approaches tend to result in more reproducible fNIRS data.^12^ Although the equipment and processes required for guided approaches may not be feasible for every fNIRS application or research study, methodical optode placement remains essential to ensure reliable fNIRS data.

A common finding among fNIRS studies across multiple fields is that group-level fNIRS test–retest reliability is consistently high.^13^^,^^16^^,^^20^ When analyzing single-subject level data, however, if short-channel correction is not applied, test–retest reliability is less reproducible than group-level data, and findings from individual analyses should be interpreted with caution.^13^^,^^16^^,^^18^ Wiggins et al.^21^ investigated the test–retest reliability of speech-evoked fNIRS responses and found that reliability for the group-level was good-to-excellent. However, on the individual level, results were highly variable, with reliability metrics being fair. It is possible that reducing the influence of physiological noise, as well as ensuring consistent placement of optodes, could improve fNIRS test–retest reliability.^19^

The purpose of this study was to investigate the group-level test–retest reliability of fNIRS signals during a passive-listening experiment across two identical recording sessions. Physiological data, including extracerebral signals collected from short channels and systemic signals collected from sensors placed on the body, were used to create multiple denoising methods. These methods were then applied to the same raw data to measure the effectiveness of physiology-signal correction on the test–retest reliability of auditory fNIRS hemodynamic response amplitude estimates. Additionally, for each subject at each session, digitized optode scalp locations were collected to investigate the impact of shifts in optode locations across sessions on within-subject changes in hemodynamic response amplitude estimates.

## 2 Materials and Methods

### 2.1 Ethical Considerations

All procedures were approved by and adhered to the ethical standards of the Purdue University Institutional Review Board (protocol no. IRB-2022-687). These procedures are in accordance with internationally accepted ethical guidelines for research in human subjects. Participants provided informed consent prior to the start of the experiment.

### 2.2 Participants

Participants were recruited through local and Purdue University community outreach as well as through flyers on Purdue’s campus. A total of 15 participants (n=11 male) 19 to 31 years old (mean = 23.47, SD = 3.72) were recruited for this study. All participants passed a hearing screening in both ears at octave frequencies between 250 and 8000 Hz at 25 dB HL. Participants were fluent in American English and Spanish. Participants were originally recruited as English–Spanish bilingual speakers for a separate study reported in Rodriguez and Shader.^22^ These participants also completed the test–retest auditory fNIRS experiment presented in the current study. All participants reported no history of hearing, cardiovascular, or neurological issues.

### 2.3 Stimuli

Stimuli were presented through bilateral ER-3C insert earphones. Stimuli were composed of concatenated sentences taken from the AzBio sentence corpora, 20 sentences from the English version^23^ and 20 from the Spanish version.^24^ Sentences were presented by either a male or female speaker. The experiment was performed using Presentation^®^ software (Version 23.0, Neurobehavioral Systems, Inc., Berkeley, California, United States). For analysis purposes, auditory-evoked activity following both English and Spanish stimuli were combined into a single “speech” condition. All participants were fluent in both languages, and there were no significant differences in cortical activity between language types. The stimuli were presented at 50 dB SPL over a loudspeaker. Speech stimuli consisted of 40 trials of two to three concatenated sentences ∼6  s in total duration. Ten control stimuli (periods of silence 6 s in duration) were also included. All stimuli were presented in a randomized order with an inter-stimuli interval (ISI) of 15 to 30 s.

### 2.4 fNIRS and Systemic Physiological Acquisition

**Fig. 1:**
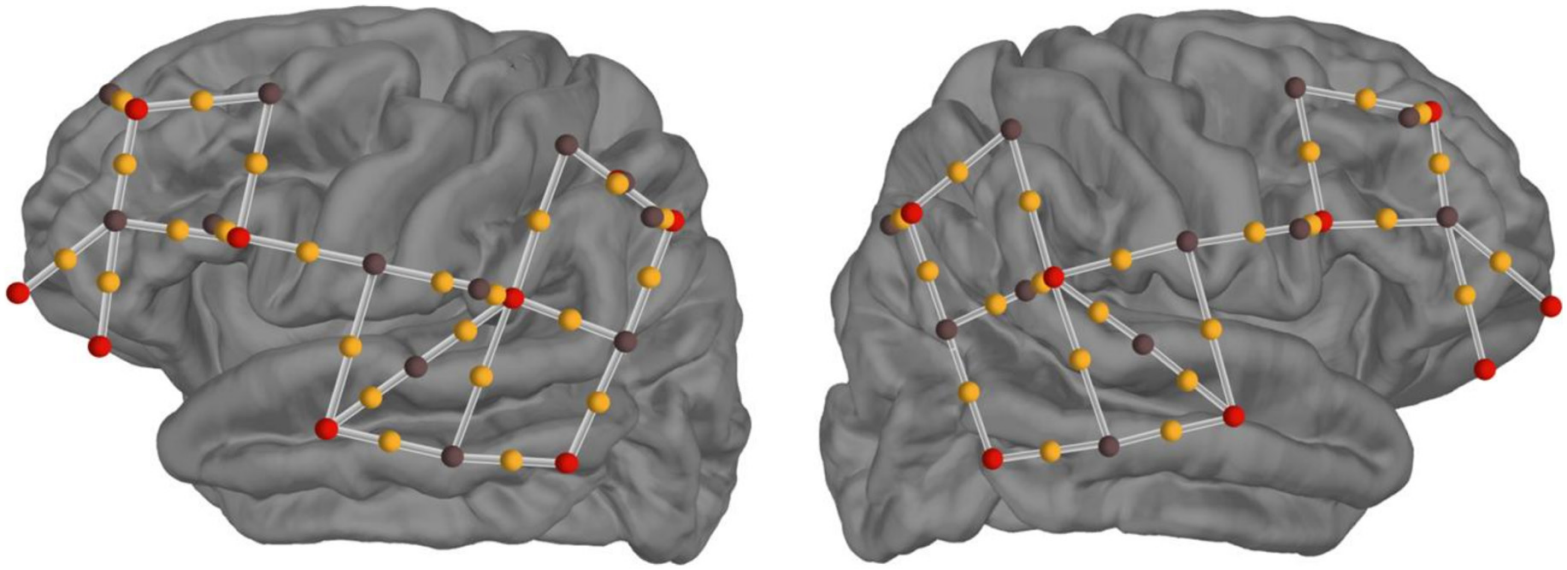
3D model of sources and detectors for the task-specific montage. Red circles indicate source locations, brown circles indicate detector locations, and yellow circles indicate channel midpoints. Short channels are indicated by overlapping brown and red circles. Optodes were placed on the left and right IFG, the left and right primary auditory cortex, and the left and right secondary auditory cortex.

Data were acquired using a portable continuous-wave NIRSport2 device and a NIRx WINGS device (NIRSport2, NIRx Medizintechnik GmbH, Berlin, Germany). The NIRSport2 device used 16 LED sources—two infrared light illuminators with wavelengths in 760 and 850 nm—and 16 avalanche photodiode detectors with a data acquisition rate of 5.1 Hz. A total of 38 source-detector pairs were placed ∼3  cm apart (Fig. 1). Sources were placed on locations AF7, F7, F3, FC5, T7, CP5, P7, P3, AF8, F8, F4, FC6, T8, CP6, P8, and P4. Detectors were placed on locations F5, FC3, C5, TP7, CP7, CP3, P5, F6, FC4, C6, TP8, CP8, CP4, and P6. Eight short-channel detectors were included in the montage to collect scalp-level extracerebral activity and were placed 8 mm from source optodes at locations F3, FC5, CP5, P3, F4, FC6, CP6, and P4. The montage *a priori* regions of interest (ROIs) were selected using the Jülich atlas provided by the fNIRS Optodes’ Location Decider (fOLD) toolbox.^25^ The selected ROIs included bilateral inferior frontal gyri (IFG), bilateral primary auditory cortex, superficial to Heschl’s gyrus, and bilateral secondary auditory cortex, including the planum temporale. Each ROI was selected for its known role in listening and speech understanding. The IFG was selected for its role in speech and sentence processing.^26^^,^^27^ The primary and secondary auditory cortices were selected for their role in auditory information processing.^28^^,^^29^

The NIRxWINGS (NIRSxWINGS, NIRx Medizintechnik GmbH, Berlin, Germany) device was used to gather systemic physiological data that could influence the fNIRS signal. Physiology metrics included pulse oximetry through photoplethysmography (PPG), heart rate, and oxygen saturation (${\mathrm{SpO}}_{2}$) measured via a pulse oximeter clipped to the ear lobe. Body temperature was measured by attaching a temperature probe to the participant’s palm. Galvanic skin response (GSR) was measured by attaching a GSR probe to the participant’s contralateral palm to measure changes in skin conductance. Temperature and GSR were recorded to measure response to stress levels regulated by the autonomic nervous system.^11^^,^^30^^,^^31^ Respiration was measured through the use of a bioimpedance-based respiration monitor placed below the participants’clavicles, which detects changes in the electrical impedance from chest expansion and lung volume while breathing. Heart rate, ${\mathrm{SpO}}_{2}$, and respiration metrics were used to measure changes in the cardiovascular system following stimulus presentation.^11^^,^^32^ Both the NIRxSport2 device and NIRxWINGS system were linked through the NIRx recording software (Aurora version 2023.2) to allow for integration of stimulus triggers concurrently into both the fNIRS recording and physiological recording.

### 2.5 Optode Location Digitization

The scalp locations of all source and detector optodes were digitized into Montreal Neurological Institute (MNI) coordinates for all participants prior to recording in both Session 1 and Session 2.^33^^,^^34^ This allowed for the tracking of optode placement within subjects across testing sessions. Digitized head scans were collected using a Structure Sensor Pro scanner (Structure, Boulder, Colorado, United States) attached to a seventh-generation iPad. Stickers were placed on the participants’ heads to mark five fiducial landmarks in the 3D scan: the inion (Iz), the midline central (Cz), the nasion (Nz), the right preauricular (RPA), and the left preauricular (LPA). Following completion, scans were then processed using the MATLAB FieldTrip toolbox.^35^ In addition, MNI coordinates were generated for the midpoint of each source-detector pair (channel).

### 2.6 Procedure

Each participant completed two identical testing sessions. During each session, participants were seated 1 m directly in front of a monitor screen in a sound-attenuating booth. Participants were instructed to sit quietly and to listen to sentences. Speech stimuli (N=40 trials) and silence stimuli (N=10 trials) were presented in a block-design paradigm with a randomized order for a total recording time of ∼28  min. An identical second recording session was scheduled within 1 week and during the same time of day as Session 1 (either morning or afternoon). This was done to account for natural changes in the body physiology that may occur over the span of the day and to maintain consistency in base levels of physiological activity.^12^

### 2.7 Data Analysis

All fNIRS and physiological data were analyzed within their respective sessions. Data were processed using the Python (version 3.12) MNE toolbox (version 1.71) for neuroimaging data analysis.^36^ Two separate analyses were performed: block-averaging and generalized linear modeling (GLM). Separate GLM analyses were conducted for each denoising model: No Correction, Short-Channel Correction, and Short-Channel + temporally embedded canonical correlation analysis (tCCA) Correction. Cortical activation was quantified as the hemodynamic response amplitude estimates (beta values) for each ROI and for both chromophore conditions: oxygenated hemoglobin (HbO) and deoxygenated hemoglobin (HbR).

#### 2.7.1 General linear modeling

**Fig. 2:**
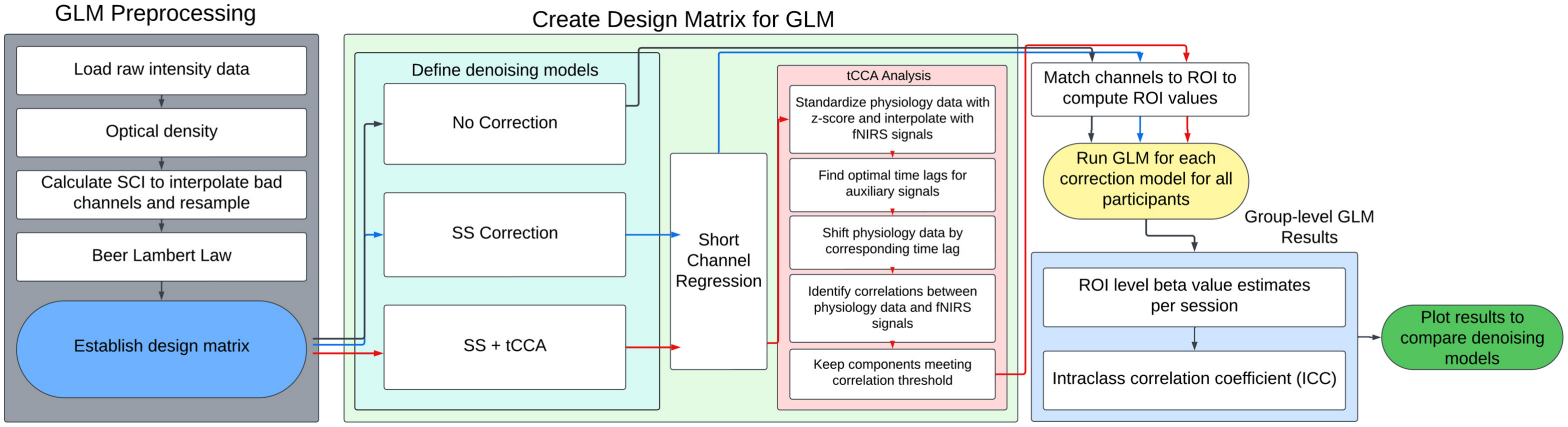
Group-level GLM analysis pipeline with denoising models for design matrix regressors. All denoising methods consisted of the same preprocessing steps. The large green box specifies each step for each denoising model included in this study. After the design matrix was created, each denoising model then followed the same protocol for group-level data processing.

GLM preprocessing, design matrix, and group-level analysis are shown in Fig. 2. Data were converted from raw data to optical density, after which the SCI threshold of 0.7 was used to remove channels that were not adequately coupled to the scalp. Data were then converted to hemoglobin concentration through the modified Beer–Lambert law before being down-sampled to 0.6 Hz.^4^ Next, a GLM of the canonical SPM hemodynamic response model (with a boxcar duration of 6 s) was used to fit the fNIRS hemodynamic responses following stimuli presentation. To perform this function, a design matrix was established to account for the timing of stimulus events and corresponding changes in the fNIRS signal. A cosine drift model with a high-pass filter of 0.015 Hz was applied to account for low-frequency oscillations and drifts in the data.

To assess the effect of different denoising strategies on fNIRS test–retest reliability, multiple denoising methodologies were established. Each method incorporated the same regressors of interest (speech and silence stimulus types) along with a constant term to account for the mean signal level, but included different configurations of nuisance regressors. The first denoising method did not include any nuisance regressors and was termed “No Correction.” The No Correction model served as a comparison to all other denoising models to assess test–retest reliability across models. A short-channel correction model (SS Correction) incorporated the mean of all short-separation channels as nuisance regressors to account for extracerebral noise originating from the scalp for both HbO and HbR hemodynamic responses. The final denoising model incorporated both short-separation channel signals and systemic-physiology signals collected from the body sensors to accomplish a short-channel correction + tCCA model (SS + tCCA).^18^^,^^37^ This method used a tCCA component to identify shared patterns of variance between the time-lagged physiological signals and the fNIRS signal. All physiology signals were standardized with a z-score, down-sampled, and interpolated to align the physiological time-series with the fNIRS signal time-series. In addition, physiology signals were subject to a low-pass filter set to a cutoff frequency of 0.5 Hz prior to the CCA step. The tCCA model incorporated unique time lags for each physiology signal, allowing for temporal variation in the time between a change in physiology and its impact on neuronal activity post-event. These lags can be used to identify each sensor’s most prominent changes in physiological activity that correlate with changes in each fNIRS channel, more accurately modeling the relationship between neuronal activity and changes in systemic physiology.^38^ Optimal time lags were selected for each combination of individual fNIRS channels and physiology signal up to 10 s following stimulus presentation to capture the peak of the hemodynamic response.^14^^,^^37^^,^^39^^,^^40^ See Sinfield et al.^14^ for a more in-depth description of each denoising method.

#### 2.7.2 Statistical analyses

Channels were assigned to specific ROIs as specified in the fNIRS cap montage (Fig. 1). ROI-level weighted averages of beta values were then calculated from weights equal to the inverse of each channel’s standard error of the GLM fit.^41^^,^^42^ A linear mixed effects model was generated to assess the group-level response amplitude estimate (beta values) for each ROI, listening condition, and chromophore.

Reliability of the fNIRS data was further compared across sessions within each denoising model by calculating the intraclass correlation coefficient (ICC). This was done to compare the group-level reliability of the task across sessions for different types of denoising methods. An ICC3 class was chosen to conduct a two-way mixed-effect model to compare the reliability of HbO estimates across sessions.^43^ This model assigned random ratings (beta values) associated with individual fixed targets (ROIs) to assess reliability across raters (sessions). ICC values were calculated for HbO response estimates across sessions in each ROI for each of the three denoising models.

#### 2.7.3 Optode placement tracking

Optode coordinates specific to each participant were extracted from the digitized scan files through the FieldTrip toolbox in MATLAB.^35^ Channels constructed from the midpoint of each source-detector optode pair were also registered to the FieldTrip MNI atlas. These channels were then classified using a corresponding anatomical label from the automated anatomical labeling (AAL) atlas.^44^

The tracking and plotting of optode location, channel location, and the shift in those locations across sessions (expressed in Euclidean distance) were completed using Python (version 3.12) and RStudio (version 4.4.1). The MNI X, Y, and Z coordinates for each optode per participant were averaged to calculate the average optode placement in each plane. Standard deviation across the X, Y, and Z planes for optode location was calculated based on the methods used for MNI coordinates of Singh et al.^45^ Values for the average optode X, Y, and Z coordinates as well as MNI coordinate sample standard deviation, calculated with Eq. (1), for both sessions are listed in Table S4 in the Supplementary Material.

$$SD=\sqrt{\left(\sigma_{x}^{2},\sigma_{y}^{2},\sigma_{z}^{2}\right)}.$$ (1)

The MNI X, Y, and Z coordinates of each optode as well as channel midpoints of Session 1 and Session 2 were used to assess shifts in both optode and channel location across sessions. This shift assessment was completed with the Euclidean distance formula shown in Eq. (2) to account for a point’s migration within 3D space. Optode Euclidean distances are listed in Table S5 in the Supplementary Material.

$$D=\sqrt{(x_{2}-x_{1}{)}^{2}+(y_{2}-y_{1}{)}^{2}+(z_{2}-z_{1}{)}^{2}}.$$ (2)

The Euclidean distance for channels across sessions was calculated for each channel per participant. Channel Euclidean distances are listed in Table S6 in the Supplementary Material. The absolute difference between the GLM-derived response amplitude estimates (beta values) for single channels between Sessions 1 and 2 was then compared with the amount of deviation in the channels’ scalp location for all channels in every participant.

## 3 Results

### 3.1 Test–Retest Paradigm

GLM for each session and for each denoising model (No Correction, SS Correction, and SS + tCCA) was performed to assess the effect of each model on the reliability of the group-level hemodynamic response elicited by speech stimuli. Statistical significance for each ROI-level beta value in Sessions 1 and 2 for both stimulus conditions was calculated through a linear mixed effects model. The resulting response amplitude estimates (beta values) for each denoising model were compared across sessions using the ICC metric to assess reliability at the group level. Block-averaged time series data for both speech and silence stimuli were created to visualize hemodynamic response morphology and are displayed in Fig. S1 in the Supplementary Material.

The results of the linear mixed effects model for each session, each denoising method, and both HbO and HbR are displayed in Tables S1–S3 in the Supplementary Material. The following statistics are reported for HbO beta response amplitudes only. For the No Correction model, significant negative (inverse) speech-evoked activity was identified in Session 1 in the IFG (β=−1.701, p<0.012) and secondary auditory cortex (β=−2.814, p<0.001) and Session 2 in the IFG (β=−2.043, p<0.005) and secondary auditory cortex (β=−2.422, p=0.001). For the SS Correction model, significant positive speech-evoked activity was identified in the primary auditory cortex in Session 1 (β=2.476, p<0.001) and also in Session 2 (β=1.315, p=0.02). Similarly, for the SS + tCCA model, significant positive speech-evoked activity was identified in the primary auditory cortex in Session 1 (β=2.6, p<0.001) and Session 2 (β=1.855, p=0.001).

**Fig. 3:**
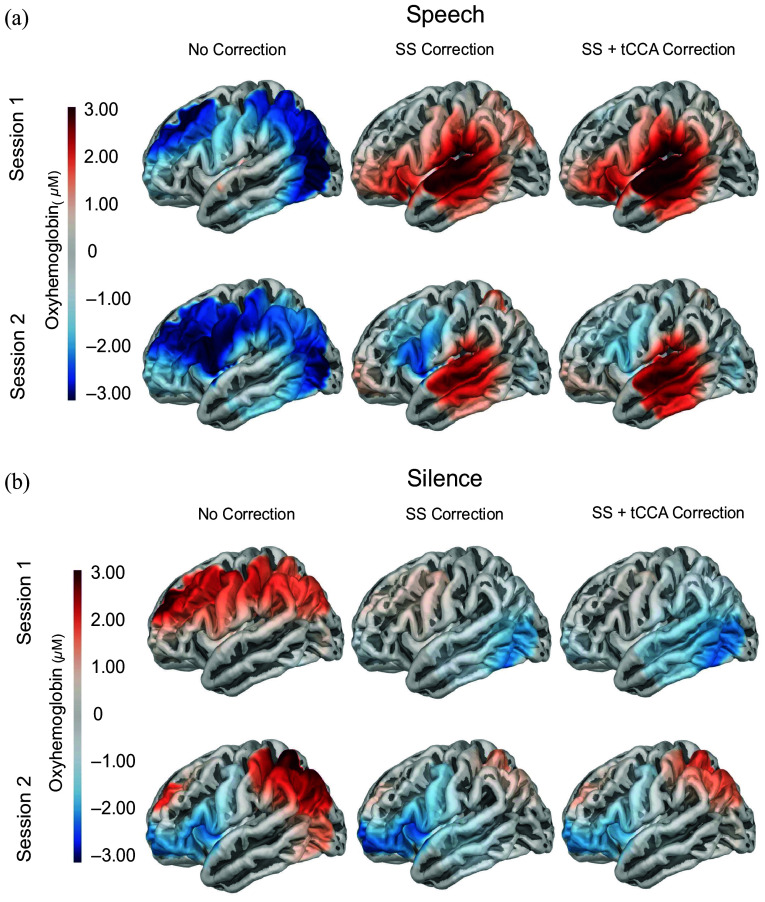
Cortical projections of beta response amplitudes are displayed for each denoising model: No Correction, SS Correction, and SS + tCCA Correction. These projections are group-level beta values resulting from the linear mixed effect models listed in Tables S1–S3 in the Supplementary Material. Each model shows the activation patterns of the left hemisphere during speech conditions (a) and silence conditions (b). Session 1 data are shown in the top row in each panel within each stimulus condition, and Session 2 data are shown in the bottom row of each panel. Activation is measured by changes in oxyhemoglobin (μM) following stimulus presentation. Red, positive values are associated with greater positive HbO concentration change. Blue, negative values are associated with greater negative (inverse) HbO concentration change.

Cortical activation plots (Fig. 3) were generated to visualize overall HbO concentration change in both sessions for speech [Fig. 3(a)] and silent (control) [Fig. 3(b)] conditions for each of the three denoising models. For the No Correction model, the speech condition elicited negative (inverse) HbO activity in both sessions, whereas the silent (control) condition elicited more positive HbO activity in both sessions. Activation during the control condition was considered a false positive resulting from physiological noise that was not accounted for in the No Correction model. For the SS Correction model, the speech condition elicited positive HbO activity isolated to the primary auditory cortex in both sessions. Significant negative activity was present in the IFG in the silent (control) condition during Session 2, which likely reflects a false positive. Finally, for the SS + tCCA model, the speech condition elicited slightly stronger, positive HbO activity in the primary auditory cortex. No substantial activity was present in the silence condition in either session for this denoising model, which differs from the SS Correction model. This effectively shows that the addition of the tCCA component in the SS + tCCA model better accounted for physiological noise and revealed less false positives in the silence condition.

**Fig. 4:**
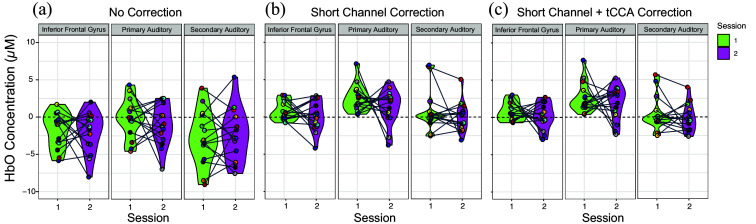
Average ROI-level beta response estimates for speech stimuli are plotted for each denoising model [(a) No Correction, (b) SS Correction, and (c) SS + tCCA Correction] for both Session 1 (green) and Session 2 (purple). Group-level data variance is represented by the distribution of the violin plots. Individual data points represent individual participant averages of all beta response estimates in the speech condition for HbO values. Individual participants are identified on each plot by a corresponding color. Participant data are linked across sessions by a grey line. ROIs for each denoising model are ordered as follows: inferior frontal gyrus, primary auditory cortex, and secondary auditory cortex.

To assess the reliability of neural response amplitudes for group-level data, Fig. 4 displays violin plots representing the distribution of individual HbO beta values elicited by the speech condition across both sessions for each denoising model. When the No Correction model [Fig. 4(a)] was applied, the resulting estimates demonstrated substantial individual variability within both Session 1 and Session 2 as well as across both sessions. In the SS Correction model [Fig. 4(b)], individual participant data showed less variability overall, with smaller group-level distributions and greater consistency across sessions for the majority of participants. Finally, in the SS + tCCA Correction model [Fig. 4(c)], there were similarly a reduction in variability within each session and a greater consistency in individual-level estimates across sessions compared with the No Correction model. Both the SS Correction and SS + tCCA models demonstrated a general decrease in variability of beta response estimates both within- and across-sessions compared with the No Correction model.

### 3.3 ICC Reliability Calculation

To assess the group-level test–retest reliability of the HbO estimates for each denoising method, the intraclass correlation coefficient (ICC) was calculated. Each ICC value was categorized into one of four categories: poor reliability (ICC < 0.40), fair reliability (ICC 0.40 to 0.59), good reliability (ICC 0.60 to 0.74), and excellent reliability (ICC > 0.75).^43^ All three denoising models produced ICC values in the excellent range: 0.835 with No Correction, 0.950 with SS Correction, and 0.997 with SS + tCCA. Of the three models, the SS + tCCA model produced the highest reliability rating. To identify the physiology signals that were most influential to the tCCA model, we examined the cross-correlation values between each physiological regressor and the fNIRS signal. An inclusion criterion of 0.3 was selected as the minimum correlation between the physiology signal and the fNIRS signal to be incorporated into the tCCA model, consistent with recommendations of Von Lühmann et al.^37^ Systemic fluctuations related to respiration and body temperature far exceeded the inclusion threshold of 0.3, demonstrating the highest correlations with the fNIRS signal. Fluctuations in heart rate and GSR signals showed moderate correlations with the fNIRS signal. Finally, ${\mathrm{SpO}}_{2}$ and PPG exhibited the lowest correlation with the fNIRS signal, despite still meeting the minimum inclusion correlation threshold of 0.3, suggesting that ${\mathrm{SpO}}_{2}$ and PPG contributed the least amount to the final tCCA components. This suggests that accounting for both extracerebral and full-body systemic-physiological signals modestly improved test–retest reliability at the group level.

### 3.4 MNI Coordinates

**Fig. 5:**
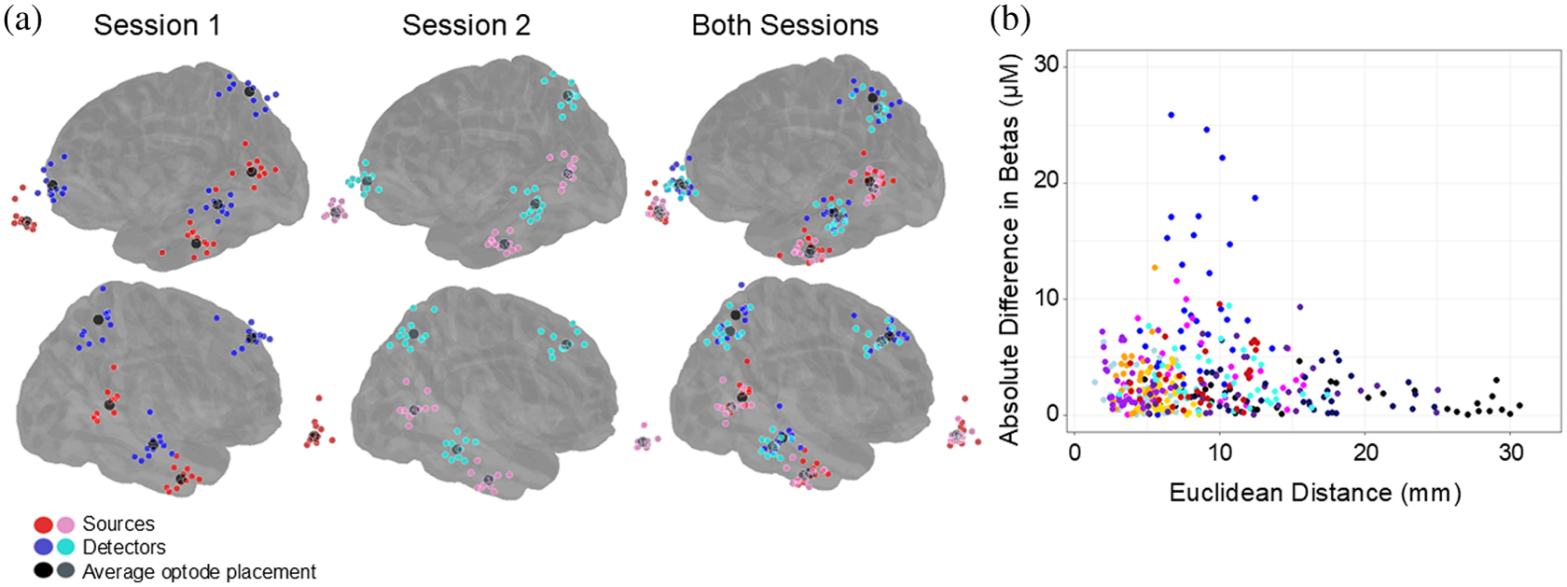
(a) MNI coordinates for three sources and three detectors forming three separate channels from the left hemisphere and their mirrored sources and detectors from the right hemisphere were selected for visualization purposes. These were selected based on their centrality within each ROI, allowing us to display all participants’ optode placements for these channels without concern for channel or optode placement overlap. Sources are marked with a red dot for Session 1 and a pink dot for Session 2. Detectors are marked with dark blue for Session 1 and a lighter blue for Session 2. Average optode placements for sources and for detectors are calculated from the MNI coordinates for all participants. Average optode placements are marked in black for Session 1 and grey for Session 2. Both session optodes and averages are plotted together to show variation in optode placement from Session 1 to Session 2. Eleven participants completed the 3D scanning component of the study. Four participants did not complete this component as the scanner equipment malfunctioned before data collection could be completed. (b) Participant Euclidean distance was calculated from channel MNI coordinates, changing from Session 1 to Session 2. Beta response estimates for each channel across sessions were calculated with short-channel corrections to remove significant noise contamination. Channel Euclidean distance values were then plotted with the absolute change in channel-level beta estimates to assess the degree of location variation and correlation with the change in beta values. Individual channels are represented by a single dot, with the color of the dots corresponding to the individual subject. The subject-specific color in this figure is consistent with their color in Fig. 4.

MNI coordinates for all individual optodes and channel mid-point locations were collected from digitized head scans of each participant prior to each neuroimaging session. Figure 5(a) displays the scalp locations of a subset of 6 optodes (three sources and three detectors) representing one source–detector pair from each ROI from all participants. The group-level average location for each optode is also projected onto the scalp surface. The average shift in scalp location across all optodes between Session 1 and Session 2 was 9.023 mm (SD = 5.898 mm). Table S4 in the Supplementary Material shows the average MNI coordinates for each optode per session as well as the corresponding standard deviation.

The optode associated with Source-8 (placed on 10-10 location P3, designating a portion of the left secondary auditory cortex ROI) was found to have the largest variance in location across participants in both Session 1 (SD = 12.971 mm) and Session 2 (SD = 10.566 mm). The group-level average difference in optode location across sessions was also largest for source 8 with a shift of 11.502 mm (SD = 7.459 mm).

To explore the relationship between differences in optode placement and the resulting response amplitude estimates across sessions, the shift in single-channel-level optode location from Session 1 to 2 was compared to the absolute difference in single-channel response amplitude estimates (resulting from the SS + tCCA model) from Session 1 to 2. Larger values for either the distance of the single-channel location shifts or the absolute difference in beta response amplitude estimates indicate larger degrees of variation for those metrics across sessions. In Fig. 5(b), greater shifts in channel location from Session 1 to Session 2 were not correlated with greater changes in the absolute difference in single-channel response amplitude estimates. This suggested that small amounts of within-subject channel migration across sessions may not contribute to significant variability in neural response amplitude estimates.

## 4 Discussion

This study measured test–retest reliability for group-level data during a passive-listening task using multiple denoising methodologies through the use of SPA-fNIRS. Neural response amplitudes were assessed for reliability across two identical recording sessions following three separate denoising models (No Correction, SS Correction, and SS + tCCA). Optode/channel migration resulting from differences in cap placement across sessions was also assessed in relation to changes in absolute neural response amplitudes across sessions.

### 4.1 Test–Retest Reliability

The three denoising models investigated here consisted of No Correction, SS Correction, and SS + tCCA. Figure 3 demonstrates positive HbO activation during speech presentation for the SS Correction and SS + tCCA models, with negative (inverse) HbO activation during speech presentation for the No Correction model. These results reflect differences in the final response amplitude estimates derived from the same raw data following separate denoising methods. Greater (positive) HbO activation in the SS Correction and SS + tCCA models suggested that extracerebral and full-body physiological noise significantly disrupts accurate estimation of speech-evoked response amplitude. The correction for extracerebral (scalp) signals contained within the short-separation channels is especially pronounced, given the change from negative HbO estimates with the No Correction model, to positive HbO estimates with the SS Correction model. This is likely because the non-neural extracerebral signal collected by short channels tends to be suppressive in nature in response to stimuli, resulting in negative (inverse) HbO values.^46^ During a task, vasoconstriction in the scalp tissue leads to reduced blood flow, resulting in a prominent negative HbO concentration change that is isolated within the short-separation channels.^46^ This result suggests that without appropriate correction methods that account for short-separation channels, the neural signal of interest may be obscured.

Additionally, when the No Correction method was applied, individual variability in the resulting response estimates was greater both inter- and intra-session (Fig. 4). Variability in the distribution of response estimates was reduced when extracerebral signals were removed from the fNIRS signal using the SS Correction model. A previous test–retest reliability study by Novi et al.^12^ utilized short channels to remove extracerebral noise from the fNIRS signal. Mean arterial pressure (MAP) and HR were additionally captured at the beginning and end of the testing session. Although short-channel correction reduced the influence of systemic physiology on the fNIRS signal, a weak to moderate connection was identified between the variability in the fNIRS signal and fluctuations in measured global systemic physiology (HR and MAP). This result aligns with previous work detailing the importance of short-channel correction in fNIRS research, as well as providing support to our findings that short channels are a crucial step in systemic-physiology removal.^1^^,^^12^ In addition, as short channels do not completely eliminate the influence of systemic physiology on the fNIRS signal, using global systemic physiology as an additional denoising method is supported.

Capturing global systemic-physiology signals in addition to using short channels provides the opportunity to remove both extracerebral and full-body physiology from the fNIRS signal. Moreover, a highly important non-neural factor affecting cerebral blood circulation is the partial pressure of carbon dioxide in the arterial blood stream (${\mathrm{paCO}}_{2}$).^7^^,^^9^^,^^47^^,^^48^ Ventilation rates have a direct impact on ${\mathrm{paCO}}_{2}$, with increases in breathing (i.e., hyperventilation) resulting in a decrease in ${\mathrm{paCO}}_{2}$.^49^ This is particularly relevant during listening or speaking tasks because speech production modifies breathing rate, meaning that changes in ${\mathrm{paCO}}_{2}$ can mirror changes in cerebral oxygenated hemoglobin. As breathing rate increases, both ${\mathrm{paCO}}_{2}$ and cerebral oxygenated hemoglobin decrease,^47^^,^^48^ which could be misinterpreted as a change in HbO due to underlying cortical activity when instead it is due to changes in respiration. Additional global systemic-physiology signals, such as respiration and temperature, may also produce non-neural changes in HbO, highlighting the importance of accounting for multiple sources of systemic influence in the measured fNIRS signal.^7^^,^^11^

An additional (slight) reduction in variability was seen following the addition of tCCA in the denoising model compared with the SS Correction-only model (Fig. 4). This suggests that additional removal of full-body systemic physiology decreased variability compared with removal of extracerebral noise alone. This result is supported by previous literature from Von Lühmann et al.,^37^ where the addition of the tCCA component significantly reduced the influence of systemic noise on the fNIRS signal. Similarly, McLinden et al.^50^ utilized a multi-modal approach with both fNIRS and EEG to assess cortical activation associated with auditory processing.^50^ A short-channel subtraction and tCCA correction model was used to remove the possible influence of systemic physiology on the fNIRS signal. They observed that the removal of systemic noise via short channels and tCCA accounted for ∼73% of the variance ($R^{2}=0.73\pm 0.14$) observed in the long-channel fNIRS signals. These results suggest that both extracerebral and global systemic physiology are responsible for the majority of variation seen in the fNIRS signal. This result provides support for our finding that both short-channel and tCCA denoising reduced variability in the fNIRS signal.

Similar denoising methods have been used to assess test–retest reliability of fNIRS data in a passive-listening task at the single-subject level. Sinfield et al.^14^ investigated six separate denoising methods, including a No Correction, SS Correction, and SS + tCCA Correction model. Their results suggested that the subtraction of physiology signals collected through short channels and a time-lagged component for a CCA component resulted in decreased root mean squared errors in the beta response amplitude estimates at the single-subject level. However, their denoising methods that included short-channel correction, including the SS + tCCA method, had slightly lower reliability metrics compared with other methods that did not perform short-channel correction. Their results suggested that physiological noise can artificially inflate reliability metrics in the fNIRS signal and that effective physiological noise correction provided a better representation of the underlying neural signal of interest, evidenced by reduced root mean squared error of the response estimates. Artificial inflation of reliability metrics on uncorrected data could also explain why the group-level test–retest reliability was deemed in the excellent range for the No Correction data in the current study.^51^ The findings of Sinfield et al.^14^ using single-subject data further support the use of both short channels as well as tCCA in fNIRS research as a way to reduce the influence of systemic-physiological noise on the fNIRS signal.

The results from multiple denoising models, shown in Tables S1–S3 in the Supplementary Material, suggested that the inclusion of short channels was particularly impactful, as our model with short-channel correction alone improved group-level test–retest reliability from Session 1 and Session 2 (ICC: 0.950) compared with our model with no correction (ICC: 0.835). Similarly, Wyser et al.^18^ demonstrated that short-channel correction improved test–retest single-subject reliability in an fNIRS motor task from an ICC value of 0.64 with no correction, to an ICC value of 0.81 with short-channel correction. Although their study investigated test–retest reliability at the single-subject level, these results support our finding that short-channel correction improves data reliability, further reinforcing the importance of extracerebral noise correction via short channels in fNIRS research.

With the inclusion of full-body systemic physiological correction through a tCCA element, group-level test–retest reliability improved slightly (ICC: 0.997) compared with correcting for short channels alone. Denoising models using tCCA specifically address the issue of fitting noninstantaneous physiology signals to the fNIRS signal. The inclusion of temporal lags allows for the identification of optimal lags in which each physiology signal is maximally correlated with changes in the fNIRS signal.^39^ Utilizing lagged physiology signals achieves the temporal component of tCCA as it accounts for the time delay among stimulus presentation, the neural response, and the change in physiology signals.^40^ Overall, each denoising model achieved excellent ICC test–retest reliability values, supporting the general reliability of group-level fNIRS. Slight improvements in reliability and reductions in variance were observed using the SS + tCCA model, which supports the future use of denoising techniques, ideally accounting for both extracerebral and full-body systemic physiology, to improve data reliability.

### 4.2 Optode Placement

The average shift in optode location among all participants across two sessions was 9.023 mm (SD = 5.898 mm). This value is similar to that of other studies that quantified optode shifts between separate sessions. For example, Dravida et al.^51^ found an average distance between the location of fNIRS source-detector channels across two sessions to be 9.5 mm (SD = 6.7 mm). In adults, this means that the variation in cap placement did not cause displacement of channels beyond the typical 30 mm channel separation. A similar study in infants conducted by Blasi et al.^52^ found that the average shift in optode location across two sessions was 2.2 mm along the x-plane and 3.1 mm along the y-plane. The authors concluded that these shifts did not have a significant impact on fNIRS data primarily because the distance of any channel shift was small relative to the typical fNIRS channel length in infants of 20 mm.

Shifts in the location of source-detector channels across sessions were not correlated with differences in response amplitude estimates across sessions [Fig. 5(b)]. Previous studies assessing group-level reliability concluded that cap removal and repositioning were associated with a sharp decrease in test–retest reliability metrics (ICC values). de Rond et al.^19^ demonstrated that fNIRS data were more reliable across multiple sessions on the same day when the fNIRS cap remained on the head in a consistent position for all sessions. Reliability then drastically reduced following cap removal and repositioning. In the current study, repeated sessions were conducted on separate days, so recapping for the second session was necessary. However, digitization procedures suggested minimal location shifts in optode positions within the same subject across sessions, and that larger shifts in optode placement were not correlated with larger across-sessions changes in fNIRS results for the same channel. In addition, ICC values across sessions also all fell within the excellent range, meaning that data were highly reliable across sessions at the group-level, especially with systemic physiological correction, despite the cap being removed between sessions.

Finally, it is important to note that individual channel-level response amplitude estimates (beta values) are not always well suited for fNIRS analysis. In most research environments, optodes are placed on the scalp of a participant’s head without any ability to assess unique participant anatomical variations of the cerebrum itself.^53^ Single-channel data is thus subject to higher levels of individual variation and, if channel-level data is analyzed individually, it may contribute to poor reliability. Using *a priori* ROI analysis procedures may help overcome this limitation. ROIs can be better defined anatomically and can provide weighted averages across multiple channels, effectively controlling for some of the variation among individual channels.

## 5 Limitations

To further investigate fNIRS test–retest reliability, a larger sample size would be beneficial. A larger sample would reduce sampling error and increase the reliability of the results, which would allow for better generalizability of our results to clinical populations. Additional testing sessions over a longer time interval would also strengthen the estimates of fNIRS test–retest reliability. This would serve as a strong model for potential uses for fNIRS in longitudinal research or clinical applications. In addition, all participants in this study had similar hair types. Including a wider range of hair thickness, length, and texture would be more representative of the general population.^54^ Scalp skin color may contribute to differences in measured hemodynamic response; a more diverse range of scalp skin color would also be more representative of the general population. Inclusion of additional physiology signals, including ${\mathrm{paCO}}_{2}$, would provide a more robust assessment of the interaction between systemic physiology and cortical activity. This is particularly important for auditory tasks, as listening and speaking may modify a participant’s breathing rate, thus affecting their ${\mathrm{paCO}}_{2}$.^47^^,^^48^ Tracking optode shifts outside of ROI boundaries may also provide more insight into the effects of differences in cap placement on hemodynamic response estimates. Future research identifying channels that migrate outside of pre-specified ROIs and analyzing channel-level data may provide further information on the effects of single-channel-level data consistency and how channel-level variations impact ROI-level analyses. Finally, participants in the current study did not provide any type of response to the speech stimuli during this passive-listening task. We cannot rule out the possibility that differences in attention or fatigue contributed to the individual variation in response amplitude estimates across sessions. Future work could evaluate the effect of systemic physiological correction on the test–retest reliability of group-level fNIRS data collected during an active-response task.

## 6 Conclusion

Extracerebral noise removal via short-channel correction improved the group-level test–retest reliability of auditory-evoked fNIRS data across two separate sessions conducted on separate days. The addition of the tCCA denoising model also resulted in excellent test–retest reliability metrics. These results provide support for applying short-channel correction in fNIRS studies to improve reliability. In addition, it is recommended that when additional systemic physiology signals are collected, a denoising model that accounts for the influence of systemic signals (e.g., SPA-fNIRS) be implemented to further improve reliability. Cap placement consistency can be quantified by measuring the movement of optodes across sessions. Small shifts in the location of the source-detector optodes across sessions did not directly influence the response amplitude estimates collected from single channels. These findings support the use of fNIRS as a reliable tool for repeated testing at the group level. This work also provides support for the SPA-fNIRS framework, suggesting that accounting for extracerebral and full-body systemic physiology improves fNIRS reliability.

## Supplementary Material

10.1117/1.NPh.13.1.015005.s01

## Data Availability

Code used for GLM analyses is publicly available on GitHub at the following URL: https://github.com/AbbieM37/SPA-fNIRS_group-level_test-retest_reliability/tree/main. All data supporting the findings of this paper are publicly available in an Open Science Framework (OSF) repository with the DOI: https://doi.org/10.17605/OSF.IO/W5C4Q.
