# Supplementary material for: Group-level test–retest reliability assessment using systemic physiology augmented functional near-infrared spectroscopy during a passive-listening task
Source: Neurophotonics. 2026 Jan 20;13(1):015005. doi: 10.1117/1.NPh.13.1.015005 (PMC12818463; doi:10.1117/1.NPh.13.1.015005)
Supplement: Supplementary file 1 [file NPh_013_015005_SD001.pdf]

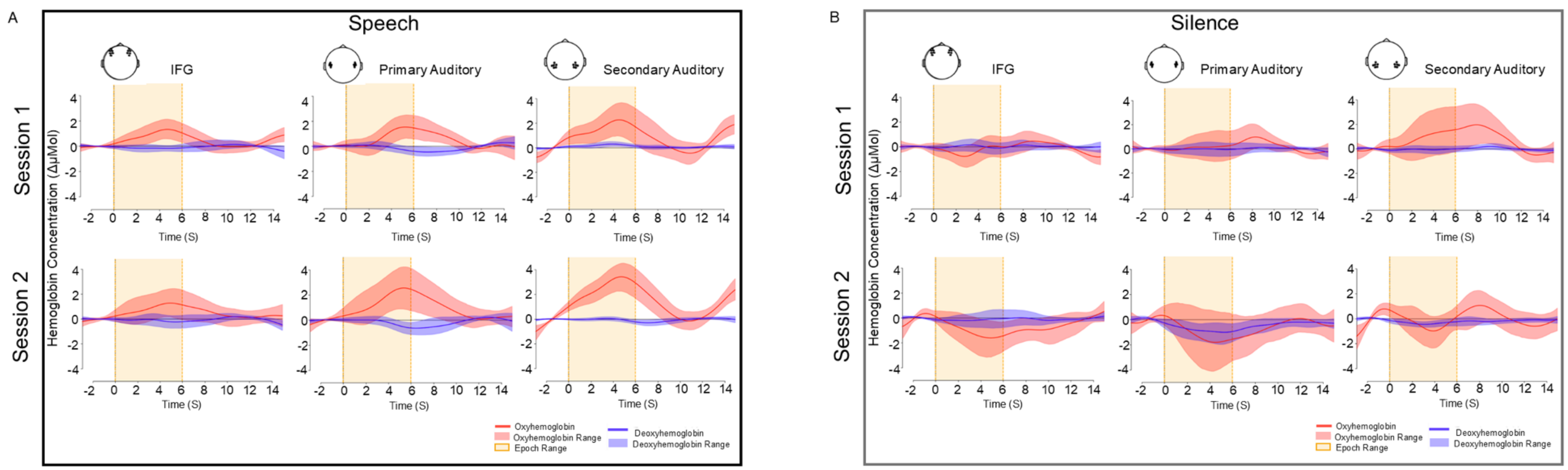

Figure S1: A block-averaging method was used to visualize the hemodynamic response morphology time-locked to stimulus onsets. Data were first converted from raw intensity data to optical density. Optodes were screened using the scalp coupling index (SCI). Any channels with an SCI below 0.7 were removed. A temporal derivative distribution repair (TDDR) correction method was applied to remove motion artifacts. Short-channel subtraction was then performed to remove extracerebral signals originating from the scalp from the long channels. Optical density data were then converted to both oxygenated and deoxygenated hemoglobin concentration using the modified Beer-Lambert Law and were then passed through a bandpass filter between 0.02 and 0.3 Hz. Epochs were then extracted at 3 sec prior to each stimulus onset to 15 sec after stimulus onset. Epochs were rejected based on the peak-to-peak amplitude exceeding 100e-6 mM to remove artifacts from data. Both HbO and HbR time series data were then plotted for speech (Panel A) and silence (Panel B) conditions in each ROI to visualize the response morphology.

Waveforms for speech stimuli were generated from 300 blocks averaged across all participants (20 speech trials per session, per participant). For silence stimuli, waveforms were generated from 150 blocks averaged across all participants (10 silence-control trials per session, per participant). Session 1 waveforms are displayed in the top row and Session 2 waveforms are displayed in the bottom row. The red line reflects the group average for HbO values, and the blue line reflects the group average for HbR values. Shaded regions surrounding the group average represent the 95% confidence interval for HbO (red) and HbR (blue). Stimuli presentation and duration are indicated by the yellow shaded region. Stimuli onset and offset are indicated by the dashed lines at t = 0 seconds and t = 6 seconds.

Table S1: Linear mixed effects model results for beta response estimates for No Correction denoising model

Session 1							Session 2						
ROI	Condition	Chroma	β	SE	z	p	ROI	Condition	Chroma	β	SE	z	p
Primary	Silence	HbO	0.604	0.919	0.893	0.372	Primary	Silence	HbO	0.419	0.974	0.573	0.567
IFG	Silence	HbO	1.042	0.919	1.540	0.124	IFG	Silence	HbO	-0.138	0.974	-0.189	0.850
**Secondary**	**Silence**	**HbO**	**1.500**	**0.919**	**2.217**	**0.027**	**Secondary**	**Silence**	**HbO**	**1.891**	**0.974**	**2.587**	**0.010**
Primary	Speech	HbO	-0.162	0.919	-0.239	0.811	Primary	Speech	HbO	-0.999	0.974	-1.366	0.172
**IFG**	**Speech**	**HbO**	**-1.701**	**0.919**	**-2.514**	**0.012**	**IFG**	**Speech**	**HbO**	**-2.043**	**0.974**	**-2.794**	**0.005**
**Secondary**	**Speech**	**HbO**	**-2.814**	**0.919**	**-4.157**	**0.000**	**Secondary**	**Speech**	**HbO**	**-2.422**	**0.974**	**-3.313**	**0.001**
Primary	Silence	HbR	0.012	0.268	0.018	0.986	Primary	Silence	HbR	-0.270	0.348	-0.369	0.712
IFG	Silence	HbR	0.159	0.268	0.235	0.814	IFG	Silence	HbR	-0.154	0.348	-0.211	0.833
Secondary	Silence	HbR	0.323	0.268	0.477	0.633	Secondary	Silence	HbR	0.236	0.348	0.322	0.747
Primary	Speech	HbR	-0.755	0.268	-1.115	0.265	Primary	Speech	HbR	-0.958	0.348	-1.310	0.190
IFG	Speech	HbR	0.027	0.268	0.040	0.968	IFG	Speech	HbR	0.334	0.348	0.457	0.647
Secondary	Speech	HbR	0.159	0.268	0.236	0.814	Secondary	Speech	HbR	0.066	0.348	0.090	0.928

Table S2: Linear mixed effects model results for beta response estimates for SS Correction denoising model

Session 1							Session 2						
ROI	Condition	Chroma	β	SE	z	p	ROI	Condition	Chroma	β	SE	z	p
Primary	Silence	HbO	-0.658	0.657	-1.303	0.192	Primary	Silence	HbO	-0.862	0.726	-1.552	0.121
IFG	Silence	HbO	-0.255	0.657	-0.505	0.614	**IFG**	**Silence**	**HbO**	**-1.169**	**0.726**	**-2.105**	**0.035**
Secondary	Silence	HbO	-0.489	0.657	-0.969	0.333	Secondary	Silence	HbO	0.470	0.726	0.846	0.398
**Primary**	**Speech**	**HbO**	**2.476**	**0.657**	**4.907**	**0.000**	**Primary**	**Speech**	**HbO**	**1.315**	**0.726**	**2.369**	**0.018**
IFG	Speech	HbO	0.851	0.657	1.687	0.092	IFG	Speech	HbO	0.190	0.726	0.343	0.732
Secondary	Speech	HbO	0.767	0.657	1.520	0.128	Secondary	Speech	HbO	0.020	0.726	0.037	0.971
Primary	Silence	HbR	0.081	0.279	0.160	0.873	Primary	Silence	HbR	-0.220	0.300	-0.397	0.691
IFG	Silence	HbR	0.270	0.279	0.535	0.592	IFG	Silence	HbR	-0.043	0.300	-0.078	0.938
Secondary	Silence	HbR	0.227	0.279	0.449	0.653	Secondary	Silence	HbR	0.228	0.300	0.411	0.681
Primary	Speech	HbR	-0.582	0.279	-1.154	0.248	**Primary**	**Speech**	**HbR**	**-1.093**	**0.300**	**-1.969**	**0.049**
IFG	Speech	HbR	-0.031	0.279	-0.062	0.950	IFG	Speech	HbR	0.227	0.300	0.409	0.683
Secondary	Speech	HbR	0.358	0.279	0.710	0.478	Secondary	Speech	HbR	-0.131	0.300	-0.237	0.813

Table S3: Linear mixed effects model results for beta response estimates for SS + tCCA denoising model

Session 1							Session 2						
ROI	Condition	Chroma	β	SE	z	p	ROI	Condition	Chroma	β	SE	z	p
Primary	Silence	HbO	-0.964	0.678	-1.882	0.060	Primary	Silence	HbO	-0.462	0.704	-0.855	0.393
IFG	Silence	HbO	-0.604	0.678	-1.180	0.238	IFG	Silence	HbO	-0.987	0.704	-1.825	0.068
Secondary	Silence	HbO	-0.768	0.678	-1.500	0.134	Secondary	Silence	HbO	0.875	0.704	1.619	0.105
**Primary**	**Speech**	**HbO**	**2.605**	**0.678**	**5.088**	**0.000**	**Primary**	**Speech**	**HbO**	**1.855**	**0.704**	**3.433**	**0.001**
IFG	Speech	HbO	0.782	0.678	1.528	0.127	IFG	Speech	HbO	0.216	0.704	0.400	0.689
Secondary	Speech	HbO	0.442	0.678	0.862	0.388	Secondary	Speech	HbO	-0.204	0.704	-0.378	0.705
Primary	Silence	HbR	-0.050	0.253	-0.098	0.922	Primary	Silence	HbR	-0.293	0.297	-0.542	0.588
IFG	Silence	HbR	0.189	0.253	0.370	0.712	IFG	Silence	HbR	-0.112	0.297	-0.207	0.836
Secondary	Silence	HbR	0.208	0.253	0.406	0.684	Secondary	Silence	HbR	0.270	0.297	0.500	0.617
Primary	Speech	HbR	-0.612	0.253	-1.196	0.232	Primary	Speech	HbR	-1.021	0.297	-1.889	0.059
IFG	Speech	HbR	-0.033	0.253	-0.064	0.949	IFG	Speech	HbR	0.270	0.297	0.499	0.618
Secondary	Speech	HbR	0.323	0.253	0.630	0.528	Secondary	Speech	HbR	-0.072	0.297	-0.134	0.894

Tables S1, S2, and S3 contain the results of linear mixed effects models on the beta response estimates from the corresponding denoising model based on ROI, stimuli condition, and chroma. Beta estimates are reported with corresponding standard error (SE), z scores, and p-value.

Table S4: Average variation in optode MNI coordinates for Session 1 and Session 2

Session 1						Session 2					
Optodes	ROI	X (mm)	Y (mm)	Z (mm)	SD across MNI (mm)	Optodes	ROI	X (mm)	Y (mm)	Z (mm)	SD across MNI (mm)
S1	IFG	-63.745	-67.417	-3.129	9.584	S1	IFG	-63.583	67.437	-2.638	6.149
S2	IFG	-82.122	40.129	-6.17	10.411	S2	IFG	-81.009	41.138	-7.274	6.285
S3	IFG	-59.303	50.589	46.257	8.913	S3	IFG	-59.262	49.961	45.049	7.876
S4	IFG	-88.844	14.557	27.207	8.841	S4	IFG	-87.795	14.657	25.982	7.106
S5	Primary	-99.063	-24.86	-4.074	9.365	S5	Primary	-97.776	-23.901	-4.644	7.253
S6	Primary	-87.219	-52.913	29.048	9.45	S6	Primary	-87.23	-53.446	27.709	8.132
S7	Secondary	-78.13	-82.695	0.091	12.215	S7	Secondary	-78.066	-85.977	-1.554	9.217
S8	Secondary	-55.655	-86.036	51.199	12.971	S8	Secondary	-56.033	-88.558	48.447	10.566
S9	IFG	66.538	63.691	-3.143	11.73	S9	IFG	66.569	63.543	-3.969	8.019
S10	IFG	83.253	35.031	-9.544	11.001	S10	IFG	83.453	33.672	-9.929	8.418
S11	IFG	62.077	47.152	46.636	11.988	S11	IFG	62.06	45.297	45.789	9.194
S12	IFG	89.576	10.21	26.385	10.356	S12	IFG	89.291	7.61	24.908	8.701
S13	Primary	97.219	-26.029	-4.079	9.198	S13	Primary	96.185	-29.028	-6.017	8.67
S14	Primary	84.291	-58.925	31.543	9.769	S14	Primary	83.373	-61.439	27.716	8.956
S15	Secondary	72.349	-89.487	0.732	10.717	S15	Secondary	71.54	-91.341	-2.362	8.189
S16	Secondary	51.214	-90.96	49.059	11.301	S16	Secondary	51.18	-93.239	45.862	9.13
D1	IFG	-76.291	46.545	19.881	9.143	D1	IFG	-75.31	46.942	18.284	7.238
D2	IFG	-70.348	19.372	57.917	8.545	D2	IFG	-70.348	18.88	55.919	7.603
D3	Primary	-92.879	-17.572	30.718	9.639	D3	Primary	-92.578	-18.971	28.286	8.345
D4	Primary	-94.669	-35.784	13.456	8.873	D4	Primary	-94.977	-36.84	13.266	8.259
D5	Primary	-92.981	-51.564	-2.928	9.368	D5	Primary	-93.149	-52.048	-5.528	6.685
D6	Secondary	-70.361	-52.61	63.456	11.646	D6	Secondary	-69.896	-53.997	62.272	9.48
D7	Secondary	-71.069	-84.661	28.41	12.781	D7	Secondary	-73.279	-85.61	26.41	9.771
D8	IFG	77.921	44.433	19.86	10.183	D8	IFG	78.033	42.392	18.631	7.654

D9	IFG	73.016	15.373	56.789	11.275	D9	IFG	73.631	12.512	54.893	9.344
D10	Primary	93.115	-23.716	30.982	10.159	D10	Primary	92.482	-26.463	27.973	9.086
D11	Primary	94.705	-39.164	13.809	9.966	D11	Primary	94.251	-42.451	10.354	8.032
D12	Primary	91.036	-56.006	-1.638	9.959	D12	Primary	89.016	-59.392	-5.186	8.496
D13	Secondary	66.228	-59.608	65.293	10.721	D13	Secondary	66.696	-62.317	60.959	9.44
D14	Secondary	66.315	-89.809	27.31	12.094	D14	Secondary	65.653	-90.503	23.946	9.148

Table S5: Average Euclidean distance for optode shift across sessions

Optode Euclidean Distance			
Optodes	ROI	Average Euclidean Distance (mm)	SD of Euclidean Distance (mm)
S1	IFG	8.264	8.384
S2	IFG	8.339	8.18
S3	IFG	7.7	8.085
S4	IFG	7.302	7.834
S5	Primary	8.524	7.776
S6	Primary	8.223	7.705
S7	Secondary	11.459	7.664
S8	Secondary	11.502	7.459
S9	IFG	8.933	7.109
S10	IFG	8.728	6.961
S11	IFG	8.811	6.875
S12	IFG	8.076	6.572
S13	Primary	9.954	6.566
S14	Primary	9.242	6.36
S15	Secondary	8.957	6.285
S16	Secondary	9.993	5.982
D1	IFG	8.089	5.312
D2	IFG	7.633	5.291
D3	Primary	8.802	5.08
D4	Primary	8.552	5.036
D5	Primary	9.195	4.768
D6	Secondary	8.534	4.66
D7	Secondary	11.188	4.524
D8	IFG	8.914	4.295
D9	IFG	8.081	3.983

D10	Primary	9.651	3.698
D11	Primary	9.203	3.53
D12	Primary	10.142	3.255
D13	Secondary	9.392	3.131
D14	Secondary	9.332	3.064

Table S6: Average Euclidean distance for channel shift across sessions

Channel Euclidean Distance		
Channel	Average Euclidean Distance (mm)	SD Euclidean Distance (mm)
S1-D1	8.176	3.458
S3-D1	7.895	2.733
S3-D2	7.666	2.923
S4-D1	7.695	3.119
S4-D2	7.467	3.034
S5-D3	8.663	3.928
S5-D4	8.538	4.069
S5-D5	8.859	3.716
S6-D3	8.512	5.051
S6-D4	8.387	4.416
S6-D5	8.709	4.206
S6-D6	8.378	5.785
S6-D7	9.705	6.652
S7-D5	10.327	4.889
S7-D7	11.323	7.165
S8-D6	10.018	6.861
S8-D7	11.345	7.797
S9-D8	8.923	4.666
S10-D8	8.821	4.859
S11-D8	8.862	5.341
S11-D9	8.446	5.75
S12-D8	8.495	5.451
S12-D9	8.079	6.211
S13-D10	9.803	7.319
S13-D11	9.578	6.859

S13-D12	10.048	7.261
S14-D10	9.447	8.052
S14-D11	9.222	7.441
S14-D12	9.692	7.897
S14-D13	9.317	7.863
S14-D14	9.287	7.863
S15-D12	9.549	7.711
S15-D14	9.145	7.681
S16-D14	9.663	7.19
